# A neurocognitive pathway for engineering artificial touch

**DOI:** 10.1126/sciadv.adq6290

**Published:** 2024-12-18

**Authors:** Ilana Nisky, Tamar R. Makin

**Affiliations:** ^1^ Department of Biomedical Engineering, Ben-Gurion University of the Negev, Beer Sheva, Israel.; ^2^ The School of Brain Sciences and Cognition, Ben-Gurion University of the Negev, Israel.; ^3^ MRC Cognition and Brain Sciences Unit, University of Cambridge, Cambridge, UK.

## Abstract

Artificial haptics has the potential to revolutionize the way we integrate physical and virtual technologies in our daily lives, with implications for teleoperation, motor skill acquisition, rehabilitation, gaming, interpersonal communication, and beyond. Here, we delve into the intricate interplay between the somatosensory system and engineered haptic inputs for perception and action. We critically examine the sensory feedback’s fidelity and the cognitive demands of interfacing with these systems. We examine how artificial touch interfaces could be redesigned to better align with human sensory, motor, and cognitive systems, emphasizing the dynamic and context-dependent nature of sensory integration. We consider the various learning processes involved in adapting to artificial haptics, highlighting the need for interfaces that support both explicit and implicit learning mechanisms. We emphasize the need for technologies that are not only physiologically biomimetic but also behaviorally and cognitively congruent with the user, affording a range of alternative solutions to users’ needs.

## Teaser

Neurocognitive congruence is key to designing artificial touch that best integrates with human perception and action.

## THE PROMISE OF ARTIFICIAL TOUCH

Imagine a world where the concept of touch is redefined, transcending the physical barriers that once limited our interactions with the environment and each other. In this world, everything is literally at your fingertips, and objects need not be physically present to be acted upon. While shopping for the perfect outfit online, you can feel the texture of each fabric as if it were right in front of you. Tapping your order on a virtual keyboard, you feel a response that mimics the satisfying resistance of real keys underneath your fingertips. Attending a conference thousands of miles away, you can feel your child’s embrace before bedtime. In this envisaged world, engineers located in different parts of the world collaborate in real time on a manufacturing process: They manipulate the components as if they were handling them in person and make intricate adjustments with precision. Even more so, the physical limitations of a person who has lost their biological arm are no longer a barrier: With a prosthetic arm that is sensitive to touch, temperature, and pressure, they engage in a cooking class, feel the texture of the dough, discern the ripeness of fruit, and adjust stove heat with a precision that rivals natural touch. These scenarios, within the domain of science fiction, are inching closer to reality, heralding a new era where our sensory experience of touch is no longer bound by physical presence.

**Fig. 1.**
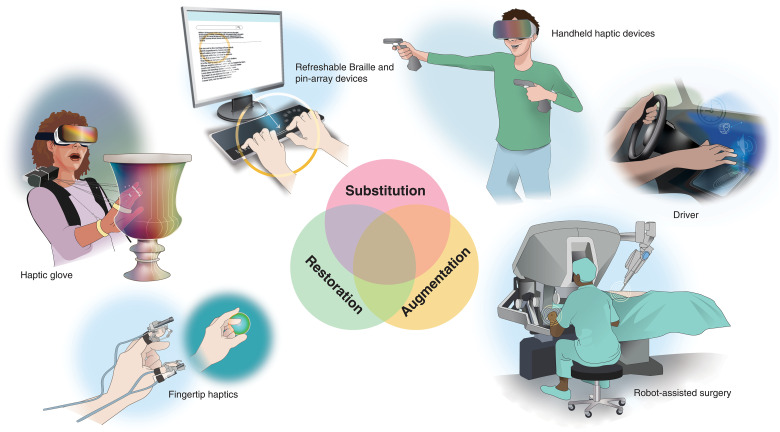
Users of artificial touch. The diverse users and applications of artificial touch technologies can be broadly categorized into three (partially overlapping) functions: substitution, restoration, and augmentation, as depicted in the central Venn diagram. Surrounding the diagram are illustrative examples for users and devices that fall within these categories. From bottom left in clockwise order: Fingertip haptics offer detailed touch feedback for virtual interactions, such as manipulating a virtual sphere, represents restoration of haptic feedback. A haptic glove for interacting with virtual art objects allows users to feel remote objects and manipulate digital artifacts as if they were real, offering restoration, as well as augmentation, of haptic feedback. Refreshable braille and pin-array devices enable visually impaired users to read digital text through tactile feedback, exemplify substitution. Handheld haptic devices for gaming enhance immersive experiences by simulating tactile interactions in virtual environments, highlighting augmentation of haptic cues. A driver using midair haptic feedback that provides tactile alerts and controls without physical contact demonstrates augmentation, as well as substitution of the touch lost because of loss of contact with physical knobs. A surgeon using a robot-assisted surgery system that enables precise control and tactile feedback during minimally invasive procedures Illustrates restoration of haptic feedback, and augmentation when the device provides assistance. For a more detailed list of related technologies, see Table 1. Illustration credit: S. Amlani.

Today’s developments in teleoperation and haptic technology are laying the groundwork for these future experiences (Fig. 1 and Table 1). In surgery, robotic systems with haptic feedback can help surgeons “feel” tissues and organs without the need to open the body of the patient or allow them to operate from afar (*1*, *2*). In manufacturing, haptic feedback can facilitate remote manipulation of materials in inaccessible environments (*3*), affording a new level of interaction in design (*4*). Touch feedback devices can aid drivers (*5*) and provide drone operators with actionable information (*6*, *7*). Haptic technologies have also been used to change the way we engage with art, by allowing audience to touch virtual art installations (*8*), and to enhance science, technology, engineering, and mathematics (STEM) education by providing interactive and tactile learning environments (*9*–*11*). Haptics also hold potential for conveying emotions through touch and enhancing emotional depth in virtual interactions (*12*, *13*). Last, assistive haptics can support individuals with disabilities. Beyond the sensory restoration of touch for individuals with a missing limb (*14*), haptic feedback devices can aid in navigating physical spaces (*15*, *16*), interacting with digital interfaces (*17*, *18*), or enhancing physical interpersonal communication (*19*, *20*) for those with sensory impairments.

**Table 1.** User cases for artificial touch. Examples of potential users who could benefit from existing artificial haptic technologies (or technologies under development). The table lists examples of artificial touch devices suitable for these users, along with the various functions these devices can provide. While these examples are not exhaustive, and there is significant overlap between the devices and their functions, this table highlights the broad applicability and potential benefits of artificial touch technology across diverse user groups. ‘V’ indicates the function(s) provided by each device. RAMIS, robot-assisted minimally-invasive surgery.

User		Device type examples	Function		
			*Substitute*	*Restore*	*Augment*
Productivity enhancement	Driver	Haptic knob, vibrating steering wheel	V		V
	Pilot	Haptic flying stick	V		V
	Drone operator	Wearable, handheld, midair	V		V
	Operation center controller	Wearable, surface, midair	V		V
	Design engineer	Externally grounded, surface		V	V
	Artist	Externally grounded, shape-changing display, digital clay, midair		V	V
	Gamer	Handheld, wearable			V
	Trainee in sports, dance, crafts, surgery	Wearable, externally grounded			V
	RAMIS surgeon	Externally grounded		V	V
	Teleoperator/tele-touch	Externally grounded		V	V
	Digital dweller	Wearable, externally grounded, handheld		V	V
	Medical simulation user	Externally grounded, shape-changing display		V	V
Individuals with somatosensory impairments	Amputation	Artificial limb	V	V	
	Patients with spinal cord injury	Haptic wheelchair		V	
	Elderly with sensory losses	Haptic belt, haptic cane	V	V	V
	Chronic stroke survivors	Wearable, haptic rehabilitation robot	V	V	V
Individuals with other sensory losses	Blindness	Refreshable braille, shape-changing, handheld	V		

Beyond the immediate interaction with virtual or remote objects, haptic technology can also enhance skill acquisition and rehabilitation. Haptic simulators are being developed to enhance the training of health care professionals by offering realistic practice environments without endangering real patients (*21*–*24*). In addition, in medical and sports training, the implementation of assistive or resistive haptic feedback is being used to shape the acquisition of skill by guiding movement and providing nuanced feedback on errors and successes (*25*–*28*). Rehabilitation, particularly following injuries or stroke, also benefits from technology that aids patients in relearning movement patterns and compensating for lost sensory feedback, thereby facilitating motor control recovery (*29*, *30*). The feedback can adapt to a patient’s progress, offering increasing resistance or assistance as recovery advances, thus providing a personalized rehabilitation journey.

These technologies, which are still very much under development, have the potential to empower users with new ways to perceive and engage with their surroundings. However, they are also slow to reach their target users and cross the chasm from the lab to real life. For example, in contrast to the picture we have just painted for the future potential of haptic feedback in surgery, its current benefits remains debated (*31*–*33*), and most clinical systems lack this feature. Similarly, while haptic simulators are being developed, they are seldom integrated into clinical curricula, and it is not clear whether training with state-of-the-art simulators generalizes to clinical proficiency (*34*, *35*). In addition, there are hurdles in bringing haptic rehabilitation out of clinical settings and into patients’ daily lives (*29*). Despite substantial advancements, the integration of haptic technology into our everyday experiences is still in its infancy. Instead, our most common encounter with haptic feedback is through the touchscreens of our smartphones. Here, simple vibrations provide a tactile response to our interactions, giving a semblance of physical touch in a digital realm. This technology extends to gaming consoles, where varying vibration patterns add a level of immersion to our gaming experiences, and to smart cars, where subtle haptic signals can alert drivers to important notifications or driving conditions. These applications, primarily based on basic vibration feedback, convey limited information and offer a rudimentary form of interaction when compared to what is envisioned. They stand in stark contrast to the wealth of sophisticated interfaces and technologies under development, which are essential for realizing the futuristic vision outlined above.

The main aim of our review is to critically evaluate this gap in innovation toward real-life applications. As our society navigates toward a reality where objects lack physical form, possibly even visual representation, the burden of conveying their essence falls increasingly upon our somatosensory system. This shift challenges our traditional understanding of objects and interaction—our somatosensory, rather than visual, system becomes the interpreter of many virtual objects. This underscores the critical need for artificial touch interfaces to provide rich yet precise and meaningful feedback to the users’ nervous system. In this review, we aim to scrutinize the feasibility of developing these complex haptic interfaces, exploring the intersection of advanced technology, physiology, and cognitive neuroscience. While previous reviews have focused on the physiological integration of artificial touch as a somatosensory signal (*14*, *36*, *37*), we will focus on how these sophisticated haptic signals could be effectively integrated with our sensory, motor, and cognitive capacities. Key to our analysis is the idea that these capacities will dynamically vary depending on our physical and mental states, contexts, and abilities. We will delve into the distinct requirements of haptics for perception versus action, requiring different device considerations. We will consider the concept of the mimetic spectrum to describe the biomimicry of the device as we transition from physiological to cognitive integration. We will highlight opportunities for nonbiomimetic design and the role of learning as a double-edged sword for the stable fidelity of haptic devices. This analysis is critical for better harnessing of the users’ brain and cognition in future artificial haptics designs. On the basis of this, we suggest that to ensure the promising potential of haptic technologies permeates our daily experiences with the digital and physical world, we might need to reconsider how to best engineer artificial touch.

Perhaps the most relevant context for artificial feedback is the control of movement. The importance of somatosensory feedback for motor control is well evidenced by the debilitating motor deficits that characterize a total loss of the ability to sense touch and proprioception, as is the case in some acute sensory neuropathies (*38*, *39*). Moreover, increasing evidence shows that the somatosensory system plays a fundamental role not only in the online planning of movement and correction processes but also in the acquisition and consolidation of motor learning (*40*–*43*). Hence, any efforts to mimic, restore, modulate, or enhance haptic information through the somatosensory system will need to be considered through the dynamic lens of the motor system. Crucially, even if the low-level (e.g., peripheral) processing of somatosensory input is similar for guiding perception and action, it will result in different high-level integration for sensations, perception, and associations, resulting in distinct behavioral, cognitive, and conceptual outcomes (Table 2). A key question that we will explore is to what extent it is important to design solutions that mirror the experiential hierarchy of the somatosensory system when developing artificial systems that are designed to feel—or function—like real touch.

**Table 2.** Levels of sensory, motor, cognitive, and emotional engagement for artificial haptic feedback. We consider seven distinct experiential categories, requiring different levels of conscious engagement. Each category enables different cognitive resources and as such can be best facilitated by distinct learning processes. It is important to emphasize that in ecological settings, these different experiential levels are much more intermixed then illustrated in the table, and as such, a given technological solution might involve multiple engagement levels. Moreover, the exact pairing between the experience and the level of awareness is state-dependent, and at times even context-dependent. Nevertheless, this table illustrates the many facets by which neurocognitive integration will be necessary to consider for effective artificial touch interfaces, emphasizing the need—and opportunities—for designs that accommodate varying levels of user awareness and cognitive processing.

Levels of awareness	Experiential level	Cognitive engagement	Learning process
Fully aware	1. How does it make me feel?	Affective	Associative
	*Subjective emotional level*		
	2. How does it feel like?	Qualia	Conceptual
	*Subjective experience of perception*		
	3. How does it inform my reasoning?	Analytical	Cognitive
	*Involves a deliberate thought process*		
Explicit	4. How does it affect my experience of the object?	Perceptual	Perceptual
	*Can be accessed by consciousness through attention*		
	5. How does it adapt my behavior (e.g., inform my actions)?	Sensory	Adaptation
	*Can be accessed by consciousness but mainly unconscious*		
Implicit	6. How does it integrate with my sensory and motor processing?	Adaptive	Habitual/Sensitization
	*Subconscious*		
Nonconscious	7. How does it make me react?	Reflex	Automatic
	*Nonconscious and automatic*		

## THE MIMETIC SPECTRUM—FROM BIOLOGICAL TO SYSTEMS-INSPIRED DESIGNS

**Fig. 2.**
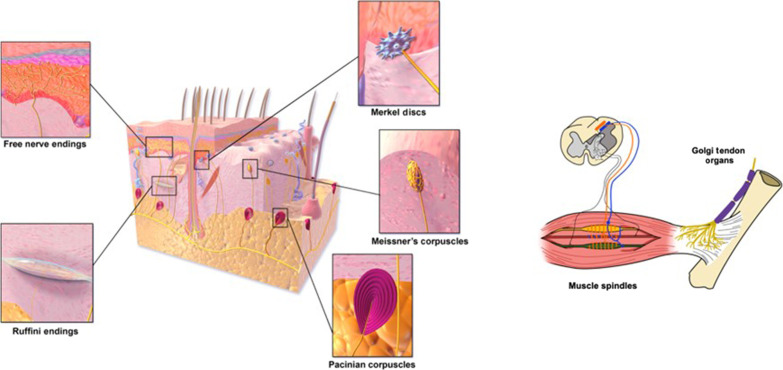
Diverse sensory receptors of the human somatosensory system to mediate haptic feedback. There is a variety of sensory receptors within the human skin and muscle structures that convey tactile and proprioceptive information. Each receptor type is specialized for detecting different aspects of sensory input. Some examples: Free nerve endings: These are unencapsulated endings, found throughout the skin and mucous membranes, that detect a wide range of sensations including pain, temperature, and itch. Merkel disks are slowly adapting receptors formed as encapsulated nerve endings in the upper layers of the skin. They respond to light touch and pressure and are crucial for detecting shapes and edges. Meissner’s corpuscles are rapidly adapting receptors formed as fluid-filled structure with flattened epithelial cells and the nerve winding its way through them and located just below the skin surface. They are mechanically coupled to the epidermis and are sensitive to light touch, slippage, and changes in texture. Ruffini endings are slowly adapting receptors formed as a cylindrical capsule with branched fibers and located in the deeper layers of the skin. They are involved in the detection of skin stretch. Pacinian corpuscles are rapidly adapting receptors formed as large, onion-shaped capsules surrounding the nerve ending located deep in the skin (and in other tissues). They are sensitive to deep pressure and vibration. Golgi tendon organs are located at the junctions between muscle and tendon; these receptors monitor tension development in muscles, providing information on the force of muscle contractions. Muscle spindles are located within the belly of muscles and primarily detect changes in muscle length and shortening velocity, hence informing about body position and velocity. The variety of receptors shown highlights the sophistication of human sensory input, underscoring the challenge of creating artificial systems that can provide comparable sensory experiences. Figure elements were adapted from Wikipedia and Wikimedia Commons under CC BY 3.0 licensing: free nerve endings, Ruffini endings, tactile corpuscle, Merkel discs from Blausen.com staff (2014). “Medical gallery of Blausen Medical 2014”. WikiJournal of Medicine 1 (2). DOI:10.15347/wjm/2014.010. ISSN 2002-4436; Pancinian corpuscle, adapted from D. Souza. Muscle spindles adapted from figure 1 in (*152*).

**Fig. 3.**
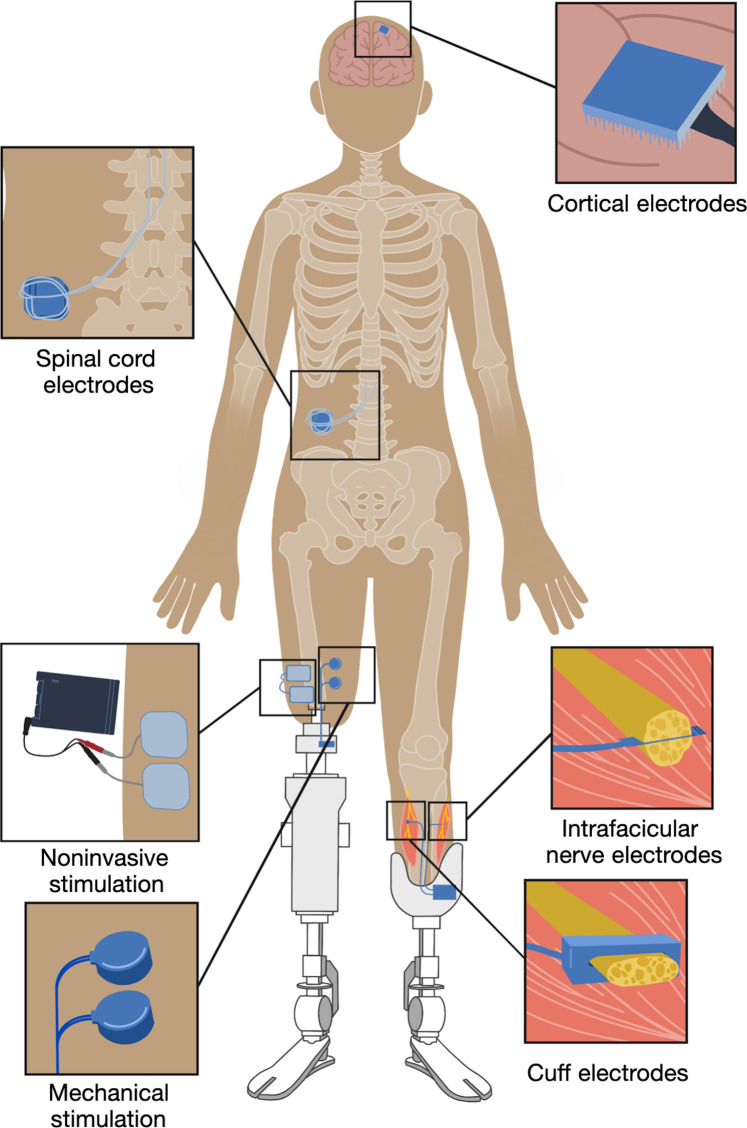
Artificial haptic feedback for prosthetic limb control in amputees. There are currently various implementations of artificial haptic feedback systems aimed at enhancing the control and sensory experience of prosthetic limbs. Most common are noninvasive actuators, such as vibrotactors, to mechanically stimulate the skin of the residual limb. Other noninvasive approaches include electrical interfaces targeting nerve fibers for sensory feedback. Surgically implanted electrodes provide direct neural stimulation, enhancing the fidelity of the feedback to mimic natural sensory inputs more closely. These include peripheral stimulation (either around the nerve or inside it), the spinal cord, and direct brain stimulation interfaces. These developments underline the availability and complexity of physiologically biomimetic interfaces. Spinal cord stimulator was adapted from (*153*) under CC BY-NC-ND 4.0 licensing.

Haptics refers to the sense of touch and proprioception, which are integral to the somatosensory system’s intricate operations (Box 1 and Fig. 2). By its definition, artificial touch is designed to interface with our somatosensory system. Let us consider one of the most mature applications of artificial touch—the restoration of haptic information for the control of an artificial limb, say for walking. Here, we want to provide the somatosensory system with the relevant information relating to the prosthetic foot, the terrane, and how these interact to influence the user’s balance—the point of contact, whether the stride is secure, and whether there needs to be a change in the control (e.g., due to slip). This information needs to be first collected using a sensor and then mediated to the user’s nervous system via an actuator or stimulator (Fig. 3). This could be done by conveying the information mechanically to the skin [e.g., of the residual limb, using a vibrotactor or a stretch of the skin (*37*)] or electrically to the nerve (*14*, *44*). Currently, most electrical neural interfaces are noninvasive, in the sense that they are not implanted. For example, many noninvasive electrical interfaces target the nerve fibers that are locally innervating the skin, or targeting the nerve more distally, via transcutaneous electrical nerve stimulation (*45*). Consequently, there is a growing interest in feeding the somatosensory system with direct electrical input, such as the surgical implantation of electrodes inside and outside the nerves (*46*, *47*), the spinal cord (*48*), and even directly to the primary somatosensory cortex (*49*). However, as the interface with the central nervous system becomes more direct, the replication of “natural” somatosensory signal patterns becomes increasingly critical. This is due to the bypassing of the skin and peripheral nervous system’s evolved abilities to convert, amplify, and filter sensory inputs.

Box 1 **Biological haptic feedback.**

The biological haptic system is a multifaceted network responsible for processing sensory information from our environment and body, encompassing various modalities including tactile, kinesthetic, proprioceptive, thermoreceptive, and pain (Fig. 2). Tactile feedback, primarily sensed by skin mechanoreceptors, enables the detection of mechanical stimuli like vibration, skin stretch, slip, and pressure, thereby providing insights into textures, weights, and the stiffness of objects (*60*). Tactile signals are thus crucial for object manipulation, offering information on the timing of physical contact, and the appropriate pressure needed for manipulation (*154*). Kinesthetic feedback, mediated by the Golgi tendon organs, adds information about the force interaction with the environment. Proprioception, primarily mediated by receptors located in muscles, tendons, and joints, informs us of our body’s position and movements within space, along with their exerted forces. The brain uses tactile, kinesthetic, and proprioceptive feedback to deduce the physical characteristics of objects through specific actions, or exploratory procedures (*155*)—for example, determining the stiffness of tissues during surgical procedures to identify malignancies through pressing (*156*), assessing the ripeness of fruit through squeezing (*98*), or estimating the contents of a box by shaking it (*157*). Haptic sensations also include additional components, such as thermosensation and pain.

In theory, if we could perfectly mimic the peripheral inputs, then the central nervous system would be able to process this artificial input in the same way it processes mechanical inputs in a natural system. The effort to mimic the biological system with high fidelity (termed biomimicry) requires particularly high precision in how the haptic signal is processed and fed back to the user, which, as we will argue below, is simply unattainable with today’s technology. Consequently, the degree to which a given technology aims to emulate the biological system—its position on the mimetic spectrum—can substantially affect how well the user’s nervous system integrates, interprets, and functionally uses the technology. Biomimicry is predicated on the (largely untested) assumption that users may be able to use biomimetic feedback to recruit preexisting neural resources, which are already specialized at interpreting somatosensory information, to support perception and action (*50*). Therefore, a successful biomimetic system will be almost immediately functionally useful, minimizing the need for device learning, and enhancing automaticity and generalization. In other words, a biomimetic approach relies on the expectation that the human somatosensory system can interpret and react to artificial input as effortlessly and naturally as it does to real, physical touch. However, this approach depends on our nervous system’s ability to effectively engage the somatosensory system to respond to the artificial touch as if it was natural. While efforts so far have been made to consider how biomimicry can be best facilitated from physiological and sensory perspectives (*14*, *36*, *37*, *51*), how to achieve biomimicry of neurocognitive processes has not been deeply considered.

From a neurocognitive perspective, biomimicry might entail some unique limitations, specifically with regards to expectations set by users’ previous experiences with natural touch. For example, biomimetic strategies might interfere with the brain’s sensory predictions based on lifelong touch experiences, potentially making it harder to integrate artificial touch seamlessly. The concept of the uncanny valley (*52*), describes an artificial representation that closely resembles the human form or sensory experiences, but is not quite accurate, consequently evoking feelings of eeriness or discomfort in the user. Paralleling this concept, attempting to mimic natural touch too closely without achieving it could lead to a mismatch between expected and delivered touch sensations, reducing the effectiveness and realism of the feedback, and even causing confusion (*53*). Artificial touch devices that aim to be strictly biomimetic might also inherit the constraints of the body, such as latency or reduced sensitivity, leading to a less efficient design. However, perhaps most importantly, strict biomimetic approaches may limit the design and functionality of artificial touch to only what is biologically familiar, potentially missing out on innovative or more effective solutions to convey haptic information.

Despite the conceptually compelling framework of the biomimetic approach, considering the high complexity of the biological somatosensory signal, other developers choose a slightly less ambitious approach, termed “bioinspired.” Here, the idea is to capture the essential qualities of the sensory signals rather than an exact replication. A bioinspired approach acknowledges that, while the sensory system can benefit from artificial inputs, these do not have to strictly emulate the natural biological processes to be effective (*54*). Instead, artificial inputs can be designed to evoke the appropriate perceptual and motor responses, based on a functional understanding of the underlying neurocognitive mechanisms. This strategy resembles how video technology simulates motion through a series of still images refreshed rapidly enough to create the illusion of continuous movement, rather than attempting to produce motion. This approach does not replicate the physiology of the system per se but rather focuses on the higher-level processing and integration of the artificial input. Current bioinspired haptic technologies do not provide their users with an analogous high-fidelity experience one might expect from the movie example. As an example in the context of prosthetic limb control: Rather than replicating the exact proprioceptive patterns that a biological foot would produce, a bioinspired system might deliver simplified cues that represent key elements that are salient for the somatosensory system but use a physiologically displaced input channel (*55*). In this example, a touch pathway is being “highjacked” to convey proprioceptive-related artificial inputs. This example demonstrates the potential for bioinspired designs to effectively integrate with the human sensory experience by approximating rather than replicating the complexity of natural sensory inputs. Thus, while the fidelity to natural sensory signals varies, both biomimetic and bioinspired designs are guided by the overarching goal of natural interface with the somatosensory system for haptic perception.

A further extension of the bioinspired route to a systems-inspired approach balances the complexity of signal reproduction with the practicality of device design and user adaptability. Current interfaces are often guided by practical considerations, including technical feasibility, cost-effectiveness, and user experience, at the expense of detachment from the biological template. Instead, the user learns to associate the artificial feedback with specific external events, such as terrains or balance states. This strategy hinges on the brain’s capacity for cognitive and sensory learning and neural plasticity, allowing individuals to interpret these cues meaningfully within the context of their motor actions. Beyond the promise of practicality, this approach has interesting potential benefits from a neurocognitive perspective. First, by creating new associations between artificial touch sensations and actions, users can develop unique and flexible responses that may not be possible with biomimetic constraints. Minimally (or non) biomimetic feedback may therefore lead to improved generalization of touch experiences across different contexts, as they are not limited by predefined biological patterns. Moreover, novel perceptual learning requires more cognitive engagement, which can lead to stronger and more robust learning of artificial touch associations (*56*, *57*). Hence, arbitrary mapping of the artificial input allows for the creation of an entirely new touch-based interface, potentially avoiding the neurocognitive limitations we highlighted above.

It is important to note that nonbiomimetic arbitrary interfaces will have their own inherent limitations, particularly with respect to how easy and intuitive it will be to learn to use them. Moreover, while a nonbiomimetic interface could afford both lower-level (adaptation) and higher-level (associative) learning, it will not likely achieve the perceptual, qualitative, and affective experience that users might seek. For example, in a recent study (*58*), a sensor attached to a prosthetic limb’s specific digit (e.g., the thumb) was paired with an electrical stimulation evoking vivid touch phantom sensations to a different digit (e.g., index finger). Despite benefiting from improved haptic ability to control the prosthetic limb, the user’s perception of the felt touch did not shift to better align with the sensor position on the artificial digit, even after a year of daily device use. This highlights that a nonbiomimetic approach is unlikely to endow the user with the full range of somatosensory experience (Table 1). Instead, it will be limited in functionality to benefit a specific design feature. A recent study, contrasting training to use a bionic hand using a biomimetic versus an arbitrary control strategy, reported that training improved motor control, reduced cognitive reliance, and increased sense of embodiment over the device (Box 2) regardless of biomimicry (*59*). While these results are confounded to the realm of motor control, they provide a useful proof of concept for the potential utility of nonbiomimetic interfaces.

Box 2 **Embodiment and self-reports.**

A related central concept in artificial haptic feedback that has become highly associated with biomimetics is of embodiment. A device is considered “embodied” if information about it is processed and used by the nervous system as information about one’s own body part, to accomplish the same function. Hence, the very definition of embodiment is associated with the principles of biomimicry. In the context of haptic device development, embodiment is both a potentially necessary condition for the effective long-term adoption of haptic technologies and a desirable outcome of engaging with them. This dynamic creates a virtuous cycle where the intuitive integration of haptic devices is thought to enhance users’ sense of embodiment, which in turn fosters a deeper connection and more seamless interaction with the technology. The allure of this cycle lies in its promise to transcend mere tools or devices, to a system perceived and experienced as a natural extension of the human body, thereby enriching our interactions with the device and enhancing our capabilities. By mimicking the way our bodies process touch and other sensory inputs, biomimetic designs are presumed to inherently promote a stronger sense of embodiment. This alignment with the natural sensory experience is often considered a necessary step toward successful integration into our perceptual and cognitive systems, enhancing both the functionality and acceptance of haptic devices (*158*). Yet, studies using nonbiomimetic devices and interfaces, such as a toe-controlled hand augmentation device (*159*) or an avatar arm that is controlled using arbitrary hand gestures (*59*), report that device usage increases perceived sense of user’s embodiment over the device. In the latter case, the nonbiomimetic control strategy resulted in similar levels of device embodiment as biomimetic control.

Embodiment is often cited as a main consideration for successful user integration. However, the methodologies used to measure such outcomes often overrely on subjective self-reports, which can be manipulated by flawed experimental designs, including contextual bias (*160*), verbal suggestion (*161*), and response compliance (*162*). For example, the phenomenon of referred sensations, famously reported by Ramachandran and colleagues (*163*), indicated that stimulating the skin of various body parts in amputees can trigger haptic sensations in their phantom limbs. This insight has inspired engineers to explore referred sensations as a natural, biomimetic interface for integrating artificial feedback in prosthetics for amputees (*164*). However, a recent study notably demonstrated that control participants, without amputations, also report similar referred sensations when subjected to comparable experimental conditions, challenging the validity of the original self-reports (*165*). This finding sharply calls into question the usage of self-reports for deriving key design interface features without the confirmation of objective evidence. Indeed, neurocognitive research shows that amputees (*166*) and expert tool users (*167*) tend to represent their artificial arm/expert tool distinctly to a biological arm relative to control participants that are not expert device/tool users. Therefore, the current evidence does not conclusively establish a link between embodiment and successful user integration.

## SOMATOSENSATION—AN ENGINEERING PERSPECTIVE

Regardless of where a given technology sits on the mimetic spectrum, artificial touch is designed to operate on (and with) the user’s somatosensory system. Hence, it is worth visiting some of the key assumptions that are shared across technological solutions relating to the sensors that are being used to pick up the relevant haptic information and the stimulators that are used to mediate this information to the user. In particular, we need to ensure that the most relevant inputs are being picked up and that those are being mediated to the user’s somatosensory system in an informative fashion.

A fundamental component of technologies aimed at providing haptic information is the ability to accurately capture relevant environmental data, which serves as the primary sensory signal. To ensure the accuracy of the data being collected and to manage the complexity of the system, sensors (Box 3) are designed to be finely tuned to detect the specific stimulus, while filtering out irrelevant noise. This tuning involves choosing filters and setting thresholds to distinguish meaningful sensory input from background interference. This focus further results from engineering constraints and the necessity to streamline data processing. This approach, however, overlooks the complexity and the holistic nature of human haptics, which processes a vast array of sensory inputs in parallel. As illustrated in Fig. 2, the somatosensory system relies on an orchestra of different receptors, continuously collecting information from the skin surface, the deep layers of the skin, the muscles, tendons, and even the bones (*60*, *61*). Different classes of receptors will be tuned to various frequencies, modalities, and pressure ranges, providing a panoramic view of a wide spectrum of haptic features. Crucially, some of these signals might not even reach conscious awareness (Table 2), yet they might contribute to the overall tactile perception (and vice versa) (*62*, *63*). A notable example is the role of the Pacinian corpuscles. These are mechanoreceptors that are sensitive to relatively high frequencies (*64*). When a localized touch occurs, it is not just the mechanoreceptors tuned to the relevant touch properties (e.g., in terms of force and touched location) that are recruited to mediate this touch; the mechanoreceptors across the hand (*65*), and even on the bone (*66*), will become stimulated as vibrations propagate through these tissues (*67*). To this effect, even if mechanosensory input from a given finger is being blocked via pharmacological anesthetics, the brain will still be able to infer that the finger has been touched (*68*). This is likely why the perception of tactile stimuli is highly robust to noise and variability (*69*). This illustrates the complex, integrated nature of touch perception, which is impossible to replicate with a narrow bandwidth of input. However, for an engineer, most of these mechanoreceptors are not sufficiently informative, in terms of signal to noise or bits of task-relevant information, to justify the computational processing time and resources required to embed them in the system.

Box 3 **Sensors for artificial haptics.**

Sensors are engineered to detect physical stimuli, such as pressure or motion, and convert them into electrical signals. In the realm of artificial haptics, these sensors are indispensable for acquiring tactile and proprioceptive information. Each sensor type uses a different physical principle to make this conversion (*168*). For example, strain gauges deform in response to force and change their resistance due to this deformation. Force sensing resistors and capacitive sensors change their resistance and capacitance, which in turn changes the electrical signal in the sensing circuit. Piezoelectric sensors respond to pressure with a release of an electrical charge. Magnetic sensors rely on detecting a change in the magnetic field. Optical sensors detect a change in the optical properties of materials such as optic fibers and interferometers by measuring the intensity, the phase, or the wavelength of light. To create high-fidelity sensing in multiple dimensions or over large areas, several sensing elements need to be arranged in specific configurations. As a result, traditionally, force sensors trade off the fidelity of force sensing with their size—they can be either high fidelity or small. Advances in materials and nanotechnology are breaking this trade-off, allowing for monitoring force with flexible and thin sensors (*169*, *170*) with minimal or no interference with touch information (*171*, *172*). Other technologies such as image processing are harnessed to create new ways of sensing force (*173*).

For proprioceptive sensing, displacement, position, velocity, and acceleration need to be sensed (*168*). Optical sensors, such as incremental encoders that can be placed at the joints of the engineered devices, are very popular because of their robustness relying on light-sensitive diodes. Magnetic sensors, such as field plates and Hall effect sensors, are another alternative, although less reliable because of their sensitivity to magnetic fields in the environment. Accelerometers and Inertial Measurement Units (IMUs) are also very popular, but they suffer from drifts because of the need to integrate acceleration signals. Ultrasonic and capacitive sensors are used to sense proximity to objects, which may be used to infer position or as a pretouch sensor. In controlled environments, external measurement via reflective markers, magnetic trackers (*174*), or markerless tracking with machine learning tools can be used (*175*). Each sensor type has specific considerations in terms of best practices in signal acquisition and processing. Even within the same type of sensor, how the information is processed may vary depending on the nature of the information and its dynamic range.

An example of the efforts to make bioinspired sensors is the development of the BioTac sensor—a multimodal sensor that provides simultaneous information about contact forces, microvibrations, and thermal fluxes, mimicking the human finger in shape and function (*176*). It mimics the finger in several aspects, such as the mechanical structure of an elastomeric skin with fingerprints-like ridges inflated by a conductive liquid over a bone-like core and leverages the deformable nature of the finger pad in its sensing mechanism, with impedance changes across multiple electrodes providing detailed information about applied forces and their sources. In addition, microvibrations that propagate as sound waves through the fluid are sensed with a pressure transducer, and thermal sensing provides temperature compensation for the fluid and sensing of the thermal properties of the touched objects (*176*). This sophisticated mechanical and electrical design is combined with machine learning methods to extract meaningful information from the sensor (*177*). Recent advances in nanotechnology and electronics allow for substantial miniaturization of sensors and the creation of matrix-like sensing structures for force (*178*, *179*) and position (*180*) and lead the way toward more biomimetic sensing in the form of electronic skin (*51*). While these and other advancements offer promise in increasing the level of sophistication of the range of input that could be captured, the challenge remains to determine which information to present to the users of artificial touch and how.

**Fig. 4.**
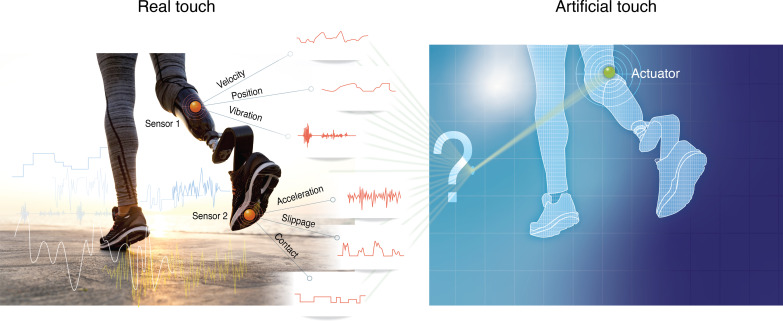
Artificial touch bottlenecks. The limited bandwidth of artificial sensors and the limited ability of artificial actuators creates bottlenecks in replicating the natural haptic feedback necessary for precise motor control. The left side shows the rich sensory data that the natural system processes, including velocity, position, vibration, acceleration, slippage, and contact information. To monitor these multiple sensory cues, multiple sensors will need to be used. The right side represents the artificial touch system, which is typically limited to one, or a few, degrees of freedom. This necessary artificial simplification entails a prioritization of specific elements of the haptic experience. This illustration highlights the difficulty in replicating complex sensory feedback. Illustration credit: S. Amlani.

The challenge of the limited bandwidth of artificial sensors becomes evident when considering complex scenarios. Take, for instance, an artificial leg encountering a slippery surface, as illustrated in Fig. 4. Determining the key haptic information to feed into the system poses substantial challenges. Is it the properties of the ground, the dynamics of the slip, or device-related aspects that are most crucial? One could consider the rate of pressure change, the angle of the prosthetic foot relative to the surface, or the force exerted during the slip, to name a few relevant signals. This choice is often arbitrary, driven either by sensor specifications or by the designer’s intuition. Even bioinspired approaches, which aim to more closely mimic human sensory processing, may not be able to fully rely on a neuroscience understanding of the somatosensory system. This is because neuroscience studies typically focus on testing theories, often using reductionist approaches, simplified laboratory experiments, or the use of animal models for answering specific questions (*70*, *71*). All these may not directly translate to practical design specifications for devices for human users. To fill in this empirical gap between the theoretical ideal of biomimetic touch and the practical limitations of current technology, engineers will have to rely on computational inferences, which are difficult to validate in humans perceptually (more on the limitations of perceptual threshold below). With our limited understanding of the human somatosensory system, the concept of biomimetic artificial touch is perhaps even more distant than the developers in the field have been acknowledging (*47*, *72*, *73*).

The issues relating to artificial sensors become even more challenging when considering artificial stimulators in haptic systems, and in particular any system that does not directly interface with the nervous system biomimetically. Stimulators, such as vibrotactile motors (*74*), skin stretch tactors (*75*–*77*), electrostimulation pads (*78*–*80*), or pneumatic bladders (*81*, *82*), are designed to convert electrical signals back into physical stimuli, essentially “informing” the user about external haptic events. These devices, while effective in generating basic sensations like vibration, skin deformation, or pressure, often struggle to replicate the nuanced aspects of natural touch. For example, a vibrotactile motor can signal the presence of an object or a surface texture change, but it may not accurately convey the object’s detailed contours or consistency. Moreover, wearable haptic devices need to be compact and lightweight enough for practical use, like in a haptic glove or a wearable sleeve, without causing discomfort or restricting movement (*83*). Devices that offer feedback beyond simple vibrations need to be anchored on the body outside of the targeted actuation zone. Moreover, pneumatic devices require the user to carry a substantial pressure source on their body. Alternatively, they can be grounded on an external surface such as a table, but this restricts mobility. Together with user safety and ethics considerations, especially when using electrical stimulation techniques (*80*, *84*), these constraints substantially limit the range of sensations that actuators can practically, safely and comfortably deliver.

In striving for biomimicry, current stimulator technology cannot fully replicate the depth and breadth of natural haptic feedback. Skin stretch (*75*–*77*) and pneumatic actuators (*81*, *82*) excel in simulating skin deformation and pressure but cannot replicate texture or temperature. Advances in soft robotics (*85*), artificial muscles (*86*), and materials sciences (*87*) introduce new actuators for haptics—soft materials such as gels and polymers, nanomaterials such as carbon nanotubes, nanoparticles, or nanowires, composite materials, rheological fluids, and others. However, each technology is optimized to convey a certain stimulus type while necessitating neglect of others due to manufacturing or control challenges. These novel materials paved the way for stretchable actuators (*88*) and active textiles and garments (*89*, *90*) that integrate seamlessly with movement and may cover broad areas of the body, but they tend to offer more generalized sensations, diluting the specificity of localized touch experiences. Another innovative avenue, midair haptics, uses focused ultrasonic waves or air jets to exert pressure on the skin, allowing users to “feel” virtual objects and textures without touching a physical interface (*91*, *92*). However, given the relatively faint feedback, the effectiveness of midair haptics substantially depends on the user’s cognitive ability to focus on the properties of the virtual object, as the sensations produced are quite subtle (Table 1). At the other end of the mimetic spectrum, surface haptic technologies enrich interactions with touchscreens beyond what current consumer devices offer (*93*). These devices use various actuators, such as electrostatic or ultrasonic ones, to modulate the friction of the surface and create perceivable textures or flat virtual objects. Shape-changing displays (*94*, *95*), encountered-type haptic displays (*96*), and particle jamming (*97*) are other ways of creating programmable haptic experiences by creating objects that can change their shape and mechanical properties. However, even though the latter solutions render actual objects, they are not exact replicas of the real objects and as such, interaction with these sensory cues might still require learning and adaptation (more on this below). Despite this wealth of solutions, it is important to emphasize that, no matter where they fall on the mimetic spectrum, the skin (or nerve) receives only a simplified representation of the rich, multidimensional sensory experience. This reduces the high dimensionality of natural touch to one or two dimensions of sensory experience, which can result in the loss of critical aspects of the sensory signal. As a consequence, the tactile feedback being relayed to the user is a substantially distilled version of reality. In these scenarios, it falls upon the designers to subjectively decide which dimensions of the sensory experience are most critical to convey.

Another important technological barrier that results from artificial haptic feedback systems, especially in the context of motor control, is the temporal lag inherent in the process of conveying sensory information may deem it dysfunctional. Unlike the human peripheral nervous system, which can transmit rich sensory data to the central nervous system within a remarkably swift time frame of 20 to 30 ms, artificial systems lag substantially behind in this aspect. This delay arises from multiple stages in the artificial feedback loop—filtering, processing, actuating the sensory data, and, in a nonstrictly biomimetic system, the cognitive cost of interpreting the information to indirectly inform motor control. Each stage, though crucial, adds to the overall time taken to relay information back to the user and, depending on the exact technology, delays can be up to several hundreds of milliseconds and even more. The human sensorimotor system inherently compensates for intrinsic sensory and motor delays: It combines feedforward and feedback control to cope with the internal delays and adjusts to the change in these delays throughout the course of the lifetime of an individual. However, extrinsic delays have been shown to impact perception and action. The threshold for perceptual and performance degradation in the somatosensory system is relatively low, with noticeable effects starting from delays as brief as 50 ms (*98*–*100*). These delays alter the perceived mechanical properties of objects such as stiffness (*98*, *99*, *101*, *102*) and weight (*103*), and with large enough delays, the perception of the cohesiveness of an object breaks down, affecting the user’s ability to interact effectively with their environment. Similarly, cognitive neuroscience research has long recognized that lags between motor execution and sensory feedback will lead to distortions in perceived touch, due to disruption of predictive sensing (*104*, *105*) (which we will discuss below). To some extent, the sensorimotor system can use these adaptive mechanisms to cope with external delays via motor adaptation (*106*) (*106*, *107*). Nevertheless, given that delays are inevitable in artificial systems, it is important to consider how to minimize their impact on the integration with the sensorimotor system.

## CONTEXT, DYNAMICS, AND LEARNING SHAPE THE PROCESSING OF HAPTIC FEEDBACK

Up until now, we have focused on the consequences of the artificial haptics specifications on the users’ sensation and perception in highly regulated settings. We should also consider the set of neural processes that will ultimately inform the user’s behavior concerning the artificial information provided. The ultimate success of these technologies hinges on a factor that is dynamic, time varying, and variable: the user. Indeed, somatosensory integration is not a mere sum of sensory inputs but involves intricate processing for the discrimination and interpretation of various stimuli. Unlike static engineered components, users represent a complex and inherently dynamic system, characterized by intra- and interindividual differences in perception, cognitive processing, and behavioral responses. The somatosensory system does not operate in a static mode; rather, it continuously adjusts how it filters, enhances, and attenuates signals based on the context and the state of the system itself. This adaptive quality poses a substantial challenge for the delivery of high-quality artificial haptic systems, which often cannot dynamically modulate sensory feedback in real time. Most artificial systems provide a consistent level of feedback, irrespective of the changing environmental conditions or the user’s current activity. This static approach fails to replicate the nuanced and context-dependent modulation of sensory information characteristic of the natural somatosensory system.

One immediate example to illustrate this gap between the artificial and somatosensory systems is the process of sensory adaptation. The somatosensory system is highly adaptable to recurring stimuli. For instance, the sensation of wearing new shoes fades unless they cause discomfort, illustrating how repeated or noncritical stimuli are naturally attenuated by our sensory and attentional mechanisms. This process of adaptation can happen both at the peripheral (*108*, *109*) and central (*110*) nervous systems and can further be exerted via cognitive mechanisms (*111*, *112*). Conversely, spinal gating mechanisms cause the enhancement of touch sensations mediated by Aβ fibers (*113*). This is achieved through spinal cord neuromolecular mechanisms that regulate the gating of sensory information, allowing certain stimuli to be amplified, based on the history and composition of the sensory input. Another prominent phenomenon is the dampening down of sensory inputs during movement. Sensory gating is the mechanism of supressing externally generated tactile stimuli applied to the moving limb (*114*, *115*). Somatosensory attenuation is a predictive mechanism for suppressing self-generated touch (*116*–*119*). These mechanisms may be crucial for ensuring that the somatosensory system is not overwhelmed by redundant information (*120*). These and other examples (some of which we will discuss below) illustrate the nonlinear nature of sensory integration for perception in the somatosensory system. They also highlight that the context in which the sensory information is delivered will radically change the way the information is processed by the central nervous system.

This adaptive characteristic of the somatosensory system poses a unique challenge in the context of artificial haptics. To ensure that the relevant information is well received by the user, engineers rely on above-threshold perception, to ensure that the user can distinctly feel (or at least recognize) the conveyed information. Yet, we risk overwhelming the user if we constantly bombard this system with above-perceptual-threshold haptic information. This is contrary to natural haptic experiences where we are often unaware of continuous stimuli unless they signify something critical, like the heat of a cup of tea or its potential to slip from our grasp (Table 2). This adaptation function is “built in” some of our mechanoreceptors that primarily respond to changes in the somatosensory input (*61*). Continuously providing detailed haptic feedback in artificial systems could lead to sensory overload or desensitization, where the user might start to ignore the feedback over time, diminishing its effectiveness and purpose. In this context, it is important to note that perceptual thresholds in themselves are dynamic, for example, because of experience and the process of perceptual learning (*121*).

Beyond its pivotal role in shaping perceptual experiences, the arguably most critical function of touch lies in the dynamic control of action. This function is important for our discussion at this point because it is arguably the most important example of the contextually dynamic behavior of the somatosensory system. Dominant theories in motor control propose that the brain uses an internal forward model in conjunction with an efference copy of the motor command to predict the sensory consequences of our actions (*122*, *123*). This predictive mechanism allows the system to anticipate the sensory consequences of movements and control actions accordingly, ensuring fluid motor control even before sensory feedback is received. It also allows for the correction of motor errors by combining sensory feedback with its prediction (*124*). Crucially, this means that the processing of sensory input during motor control is subject to contextual modulation.

To make things even more difficult on artificial haptic feedback, the forward model in itself is forged through various processes of motor learning, which is also a highly dynamic process (*125*). Such adaptivity is intuitive in the context of skill acquisition or rehabilitation, where the purpose of the sensory information is to alter the system, but in fact, the sensorimotor system implicitly adapts perception and action even when instructed otherwise (*126*, *127*). This suggests that the challenge for artificial haptic feedback lies not only in accurately mimicking natural sensory input but also in the nuances of individual motor learning curves and the specific ways in which each user’s brain predicts and responds to sensory feedback. Recent studies have begun to unravel the complex interplay between explicit and implicit learning processes in response to altered sensory feedback (*128*, *129*), highlighting the challenge and the potential for these mechanisms to be harnessed in the processing of artificial touch. However, how to effectively shape these different learning processes to enhance the integration and utility of artificial haptic feedback is yet to be discovered.

Accounting for this adaptability in the design of artificial touch systems poses substantial technical challenges, in terms of estimating the current state of sensorimotor adaptation/learning, and in building the systems with real-time tuning mechanisms. Beyond the technical challenges, the brain’s ability to predict and attenuate self-generated sensory feedback poses a unique conceptual challenge for these systems. If the artificial feedback closely mimics natural sensations, then it might be attenuated by the brain, reducing its effectiveness. Conversely, bioinspired feedback that is distinctly different from natural sensations might not be properly integrated into the user’s motor framework. Here, we will need to consider the cognitive opportunities and costs of learning to associate a non-natural stimulus with a desired somatosensory outcome. Going back to the example of the artificial limb that is about to slip (Fig. 3)—under a systems-inspired framework that favors cognitive to biophysiological alignment, the system might be able to signal to the user an alarm cue to be mindful of a potential fall, say using a vibration of the trunk. The system might even provide information about the causes of the slip (e.g., the sensor properties of the surface and the state of the artificial limb), and how to avoid it (e.g., lean right). However, translating this artificially mediated information into effective motor decisions requires the user to engage in a complex conscious and deliberate interpretation of the artificial feedback. This involves forming a new cognitive map (*130*), where the user learns to associate non-natural stimuli, like the vibration of an alarm cue on the trunk, with specific somatosensory outcomes such as the predicted fall, to shape a motor decision. The user must not only recognize the alarm but also understand its implications—the corrective actions needed to avoid a fall. This cognitive mapping process is considerably more demanding than responding to natural somatosensory input, which often involves reflexive actions that can even be mediated at the spinal level (*131*). It will also require a deliberate learning process, during which users may need to undergo extensive training and experience numerous failures—such as falls in the case of an artificial limb—before they can accurately interpret and respond to the artificial cues. Crucially, throughout this process, the key learning mechanisms that will be used are not specific to the somatosensory system, meaning that the artificial haptic system will not necessarily be integrated as such. During this cognitively costly learning phase, the artificial feedback becomes a source of information that the user gradually learns to integrate into their cognitive and motor frameworks.

It is also important to highlight that the unique challenges of integrating haptic feedback for motor control may be entirely distinct from other applications for haptic feedback that are geared toward perception and affectation. The processing of sensory information by the brain is markedly different when it is intended for perceptual understanding compared to when it is used for guiding actions. In the visual system, this is described by the dual-pathway hypothesis, where the dorsal stream processes visual information for action while the ventral stream is more involved in perception and recognition (*132*). A similar distinction between the processing of information depending on whether it is intended for perception or action extends also to the somatosensory system. An illustration of this is the size-weight illusion, where larger objects that weigh the same as smaller ones are perceived lighter upon lifting (*133*). This illusion has been shown to link to our haptic experience (*134*), and it also affects how we adjust our grip strength as we anticipate the different weights. However, within a few lifts, motor learning enables us to adjust the grip to the true weight, despite the ongoing perceptual illusion (*135*). Likewise, delayed haptic feedback changes our perception of the stiffness of elastic objects, making them feel softer. Yet, through repeated interactions, we learn to adjust our grip force accurately, timing it appropriately and calibrating it to the actual stiffness, despite the ongoing perceptual illusion (*101*, *136*). These examples highlight two important properties of the differing demands placed on somatosensory processing for perception and action. First, somatosensory processing for perception is less reliable (in terms of object representation) than for action. Second, somatosensory processing for action compensates for inaccuracies via motor learning and adaptation.

The distinct processing for perception and action adds complexity to integrating artificial feedback with the somatosensory and motor systems. However, it also presents a chance to explore flexibility in design that current haptic technologies miss: optimizing perception and action separately. For instance, designers of teleoperation systems with haptic feedback typically prioritize the transparency of the system, seeking to match the movement and force between the local and remote manipulators while overlooking the human user. Yet, this goal can clash with system stability—its ability to operate without oscillations, erratic behavior, or uncontrolled growth of signals (*137*). From a user perspective, a better approach might involve designing controllers that correct perceptual distortions at the user’s end and optimize action at the remote site, offering a setup that is perhaps not transparent but supports natural perception and action more effectively (*138*).

## THE FUTURE SHOULD BELONG TO EVERYONE: EMBRACING DIVERSITY IN HAPTIC DESIGN

As we advance toward integrating haptic feedback systems into our daily lives, it is imperative to acknowledge the spectrum of diversity among potential future users. For haptic technologies specifically, diversity refers to people with a wide range of body types and a range of cognitive and physical abilities. These varying abilities might relate to age, gender, weight, lifestyle, range of physical and/or cognitive ability and disability, and people’s cultural, financial, and societal preferences. For example, aging involves degradation of mechanoreceptors and the thinning of skin, which will markedly change people’s tactile thresholds and ability to wear and tolerate haptic feedback. Something as simple as the size of the fingertips or other body parts can affect the effectiveness of haptic stimulation (*139*). Body fat distribution, which will vary on the basis of gender and lifestyle, can substantially influence how haptic signals are perceived, as different levels of subcutaneous fat can alter the intensity and quality of tactile feedback. Similarly, ethnic differences can affect skin properties, such as thickness and elasticity, which may in turn affect the efficacy and comfort of wearable haptic devices. In addition, cultural differences may shape preferences for the type of haptic feedback, where on the body it is applied, and at what intensities it may be deemed acceptable or preferable, further underscoring the need for a culturally sensitive approach to the design and implementation of haptic technologies. Even for the same person, the cognitive and emotional state may change the efficacy, comfort, and acceptability (*140*). Designing with these factors in mind ensures that haptic technologies are accessible and enjoyable for a broader user base. Moreover, these considerations are also tightly connected to both ethical concerns and broader social implications of haptic technology (*13*)—for example, the potential for misuse in manipulating or deceiving people (*141*) and the need to ensure that the technology is developed and deployed in a way that respects user consent and autonomy. Neurally invasive haptic interfaces will also need to consider the intersection between their technologies and user’s autonomy, identity, and responsibility for a brain in a neural interface (*142*).

We also want to highlight the design and societal risk of reliance on personal intuition for design solutions. Our collective intuition has often led us astray. As illustrating examples, take our discussions of embodiment and referral of touch in Box 2. Perhaps the biggest risk with letting our intuition lead our innovation is the fact that what makes sense to one person might not ring true to a different individual. In light of these considerations, our responsibility extends beyond mere compatibility testing. To ensure that research for innovation is successful, there is a need for meaningful involvement of end users in the research and development process, with the aim to improve the quality and impact of research while also emphasizing responsible research practices. This demands a commitment to inclusivity from the earliest stages of design, ensuring that haptic technologies are accessible and beneficial to a wide audience. One way to achieve this interaction is through public engagements. For example, we have recently used a science exhibition to test the wearability and use compatibility of a novel technology on a highly diverse group of 596 participants (age range of 3 to 96), demonstrating successful device use from the very first minute of experience in 98% of the cohort tested (*143*). Another promising avenue is adopting a framework for involving end users in the research and development process of technologies (“Public and Patient Involvement and Engagement,” “lived experience,” “co-design,” “co-production,” etc.) (*142*). A common feature to these different frameworks is allowing users and their stakeholders to influence and guide the decisions made about the development of technology and how it will be implemented. Integrating an end-user involvement framework as a continuous evaluation tool with bidirectional feedback throughout the research process has the potential to enhance the quality, relevance, and ethical foundation of innovative research and is expected to cause greater impact. This inclusivity not only broadens the impact of technological advancements but also ensures that the future of haptic feedback truly belongs to everyone.

## THE PATH FORWARD—HARNESSING THE NEUROCOGNITIVE CONTINUUM TO IMPROVE ARTIFICIAL HAPTIC INTERFACES

**Fig. 5.**
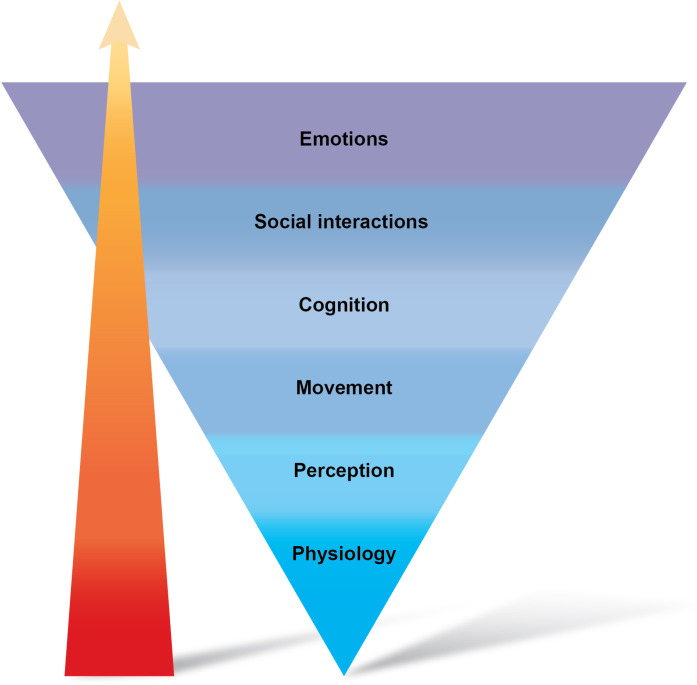
A systems approach to mimetic artificial haptics: Physiological biomimicry is just the tip of the iceberg. This figure aims to illustrate the rich behavioral context in which haptic feedback will be integrated. Currently, mimicry is most commonly explored in terms of physiological compatibility, under the assumption that high fidelity of how natural input is physiologically integrated will provide ideal infrastructure for natural integration of artificial haptics in all behaviors. However, considering the serious challenges that physiological biomimicry is currently facing, we propose to turn this hierarchy on its head—rather than its basis, physiological biomimicry is the tip of the iceberg for better integration of artificial haptics. The behavioral context (and need) for a given technology should also provide unique considerations (and opportunities) for how to best integrate artificial information with the user’s body, mind, and brain. The red arrow illustrates that designers have been paying more attention to the bottom layers of the reverse pyramid, and hence, efforts should be concentrated in exploring opportunities at the higher levels, starting with perception, movement, and cognition.

On the basis of our analysis so far, it appears that the binary of biomimicry versus nonbiomimetic designs does not necessarily capture the complexity of the challenge of interfacing artificial haptics with the user’s neurocognitive mechanisms. No matter how sophisticated the technology becomes, or how closely it mimics biological systems, its effectiveness should be determined by its contribution to cognitive, motor, and learning behaviors, as illustrated in Fig. 5. Embracing a systems approach that considers not only how the input is integrated physiologically across the somatosensory system (*144*) but also behaviorally encourages us to also study how cognitive elements can profoundly influence the effectiveness of these interfaces. We believe that, as the sophistication and dimensionality of haptic interfaces increases, the neurocognitive continuum (Table 2) offers interesting opportunities for enhancing human-machine interaction, which have been mostly neglected from the discussion of biomimicry. Ideally, these mechanisms should work in harmony to best express the key haptics considerations to the users in the most nonintrusive fashion. In this final section, we highlight some practical considerations for the successful delivery of artificial haptics at the behavioral and cognitive levels.

 In the context of cognitive compatibility, we the need to better harness and tame sensory information that is not fully consciously perceptible. As highlighted above, our somatosensory system is most powerful when it is not constantly dominating our conscious experience but rather can be highlighted and attenuated on the basis of our attentional state (see Table 2). “Calm technology” refers to a design principle that aims for technology to operate in the background of our lives, providing information or functionality without requiring focused attention. The goal is to reduce cognitive load by allowing technology to integrate into our environment without overwhelming us with information or requiring constant interaction. This approach emphasizes technology that supports our daily activities quietly, alerting us when necessary but otherwise remaining unobtrusive, thereby minimizing the cognitive demands placed on the user. It is therefore essential to balance the cognitive demands of interacting with artificial touch systems with the cognitive resources available to the user. Here, we wish to highlight opportunities of using subthreshold sensory inputs to artificial systems.

One such example is “intrinsic” feedback, a term that has been coined for incorporating natural somatosensory feedback from extra robotic body parts. Somatosensory input is inherently present at the interface between the wearable device and the user’s body. Intrinsic feedback would be any sensory information that follows from a change in the position of the device or impact between the device and environment (e.g., stretch and vibration). As this sensory information is mediated by the device, it should provide reliable information to the wearer. This information is usually at around the perceptual threshold, being barely perceptible to the user. Previous work has demonstrated that intrinsic object-impact signals during tool-use contain detailed information about the object features (*145*). It was further shown that motor control of a torso-controlled extra arm is significantly better when physically worn, compared to a virtual interface (*146*, *147*). Similarly, motor control, retention and transfer of learning, as well as performance under cognitive load were all impaired when somatosensory feedback was blocked from the toes, while learning to operate a toe-controlled robotic Third Thumb (*148*). These findings demonstrate that there are likely multiple avenues to provide somatosensory feedback that supports motor control of a wearable device, beyond artificial cues. Yet, intrinsic signals are rarely explicitly used to guide haptic interfaces. We believe that intrinsically natural interfaces hold much potential for the future of haptic feedback, and could be better harnessed, e.g., using materials that mechanically mediate the state and properties of the device with greater precision and fidelity. However, this route is limited to wearable devices and as such will not be immediately suitable for remote applications.

At the other end of cognitive compatibility, we are also inspired by how individuals learn to navigate complex environments or master video games that are relatively unintuitive. This ability suggests that with sufficient motivation and perhaps guided instructions, users could internalize the operation of conceptually novel haptic devices. This learning process, akin to forming new cognitive maps, could transform initially nonintuitive interfaces into cognitive extensions of the user’s sensory and motor systems, becoming second nature through practice, as happens when learning to drive. By focusing on the cognitive ease of integration, rather than replicating biological fidelity, we can develop technologies that are nonbiomimetic yet still user friendly. In this alternative framework, the goal should be to harness our inherent cognitive capabilities with minimal learning requirements and cognitive load. This involves designing interfaces that are intuitive and aligned with the brain’s natural processing abilities. Here, the success of the haptic interfaces in touch screens should be applauded.

We can also avoid some of the known physiological pitfalls highlighted above by tailoring the feedback to the user’s cognitive and motor states. There are several efforts to develop adaptive and customizable haptic and assistive technologies that aim to more closely mimic the dynamic responsiveness of the human somatosensory system. For example, the intensity of electrotactile feedback in a prosthesis was adjusted by measuring the electrical impedance of the electrode interface (*79*), and the assistance provided by an exoskeleton device for the ankle was adjusted by measuring the metabolic cost of walking and running (*149*). These closed-loop adjustments provided even more benefit when carefully combined with training (*150*). Although these efforts represent a promising direction, the development and integration of such adaptive systems is still at its infancy, highlighting the need for continued innovation in bridging the gap between artificial touch and the complex dynamics of human sensory processing. Here, it is important to highlight that users will also adapt to the system, leading to a mutual adaptation between the device and the sensorimotor system (*151*). Future research should explore how performance with artificial haptic systems evolves not just in controlled settings but in varied, real-world contexts, emphasizing the importance of user motivation and the potential need for targeted training to fully harness the benefits of this dynamic adaptability.

At the same time, we must also consider the substantial limitations of cognitive learning. Going back to the example of learning to drive, this is an incredibly time and effort consuming process, and the motivation for such intense learning must be justified by the benefits of the artificial system. Overreliance on cognitive adaptation for device integration could further detract from other cognitive functions, leading to fatigue or overload. We are all painfully familiar with the cognitive costs of multitasking in our daily lives, and most of us will not welcome the intrusion of yet another highly demanding cognitive task that needs to be solved in parallel to our daily activities. We need to ensure that artificial touch systems provide the same level of benefit to daily living, or else make them more naturally integrated with human cognition. Otherwise, we risk designing technology that might look promising but will eventually be abandoned. To minimize these potential cognitive side effects, future research should focus on optimizing the design of haptic devices to align with the cognitive processes involved in sensory perception and motor control. This could include adaptive interfaces that adjust on the basis of the user’s cognitive state or learning progress, reducing unnecessary cognitive effort and enhancing the user experience.

To conclude, while biomimicry of the peripheral nervous system offers unique opportunities for seamless integration with artificial haptic interfaces, it also presents enormous challenges. Integrating artificial haptic devices into our cognitive framework, without exclusive reliance on biomimicry, presents a viable additional path forward, though this path should be tread cautiously. Furthermore, different individuals may respond differently to the integration of such systems, influenced by their unique physiological, cognitive, and cultural parameters. Currently, there is a lack of evidence-based design parameters for assessing the viability of user-centered artificial touch systems using structured neurocognitive experimental manipulation. A framework for deriving design parameters for artificial touch systems will also enable hypothesis testing about the sensorimotor system. It is possible that many of the assumptions and theories guiding neurocognitive-inspired artificial touch systems are inaccurate or even false. Hence, a systematic and interdisciplinary framework will accelerate the development of innovative design strategies within and outside our biomimetic toolbox.
